# Factors associated with impaired color vision without retinopathy amongst people with type 2 diabetes mellitus: a cross-sectional study

**DOI:** 10.1186/s12902-017-0181-7

**Published:** 2017-06-02

**Authors:** N. C. Tan, W. F. Yip, S. Kallakuri, U. Sankari, Y. L. E. Koh

**Affiliations:** 1 SingHealth Polyclinics, 167, Jalan Bukit Merah, Tower 5, #15-10, 150167 Singapore, Singapore; 2 Duke NUS Medical School, Singapore, Singapore; 3 Singapore Eye Research Institute, Singapore, Singapore; 4 Optometry Centre, Singapore Polytechnic, Singapore, Singapore

**Keywords:** Color vision impairment, Diabetes mellitus, Screening

## Abstract

**Background:**

Patients with type 2 diabetes mellitus (T2DM) may develop color vision impairment. This study aimed to determine the prevalence and factors associated with impaired color vision in patients with T2DM but without diabetic retinopathy.

**Methods:**

Enrolment criteria included multi-ethnic Asian participants, age 21 to 80 years, with known T2DM for a minimum of 2 years. Their diagnoses were affirmed from oral glucose tolerance test results and they were screened for impaired color vision using the Farnsworth D-15 instrument. Demographic characteristics were described and clinical data for the preceding 2 years were analyzed using logistic regression.

**Results:**

Twenty-two percent of 849 eligible participants had impaired color vision with higher involvement of the right eye. Impaired blue-yellow color-vision(Tritanomaly) was the commonest impaired color vision. Participants with impaired color vision were significantly associated with age and lower education; longer duration of T2DM (median 6 years vs 4 years); higher HbA1c level and HDL-Cholesterol in 2nd year; lower mean total cholesterol, mean LDL-Cholesterol and mean triglyceride in 2nd year. They also have poorer vision beyond 6/12 in the affected eye. Logistic regression showed that impaired color vision was associated with older patients (OR=1.04), increased duration of T2DM (OR=1.07); prescription of Tolbutamide (OR=3.79) and lower mean systolic blood pressure (OR=0.98).

**Conclusion:**

Almost one in four participants with T2DM had impaired color vision, largely with tritanomaly. Color vision screening may be considered for participants who develop T2DM for 6 years or longer, but this requires further cost-effectiveness evaluation.

## Background

The color vision of individuals may be affected by type 2 diabetes mellitus (T2DM). This acquired form of impaired color vision (ICV) results in the inability to distinguish similar shades of a color. Deuteranopsia or deuteranomaly (less severe form) refers to red-green color deficiency. An individual with protanopsia or protanomaly (less severe form) has difficulty distinguishing different shades of the same color in the red-yellow-green spectrum. Tritanopsia or tritanomaly refers to blue-yellow color deficiency.

Amongst people with T2DM without diabetic retinopathy, the Okubo Color Study Report revealed that their adjusted odds ratio (AOR) for impaired color vision (ICV) was 5.89 compared with individuals with normal glucose levels [1]. It also reported the prevalence of ICV was higher among people with elevated LDL-Cholesterol (LDL-C) levels. Andrade et al. showed that ICV was associated with glycated hemoglobin and time of disease onset [2]. Feitosa-Santana C et al. also substantiated the correlation between ICV and the level of glycated hemoglobin [3]. They reported the presence of subclinical color vision loss in early stage of T2DM [3]. Using a different approach in assessing color vision deficiency, Sun TS and Zhang MN also found that ICV was present in early stage of T2DM and was associated with the age of participants [4]. The SNDREAMS-II Report-3 elucidated that a significant protective factor was serum high-density lipoprotein (HDL) [5].

ICV could impact on myriad daily living activities and social life of affected people, ranging from play, sports, driving, education, occupation, discrimination, health and personal safety. Trento M et al. in their multi-centered study in Italy also alluded that vision loss and ICV resulting in poor vision functioning, would impact on their social role and quality of life [6].

The prevalence of T2DM amongst the multi-ethnic Asian population in Singapore is expected to rise from 11.3% in 2010 to 15% in 2050 [7]. With its dual private-public healthcare system, 45% of these people with T2DM are managed in the local public primary care clinics (polyclinics) [8]. With increasing prevalence of T2DM, the number of people with ICV is expected to expand significantly, raising their risks of poorer quality of life and reduced social functioning. In view of findings from earlier studies [1–5], we postulated that the local patients with diabetes mellitus were at higher risks of ICV in relation to their age, duration of disease, glycaemia and dyslipidemia control. Identifying these risk factors open up a window for intervention to mitigate the development of ICV.

Thus, the study aimed to determine the factors associated with ICV without diabetic retinopathy amongst people with T2DM who were managed in primary care.

## Method

### Study site

The study was conducted in a typical polyclinic at Pasir Ris estate, which covers an area of 15 square km in the north-eastern region of the island state. This polyclinic serves an estimated population of 133,863 multi-ethnic residents in 2010, comprising of 67% Chinese, 20% Malay, 8% Indian and 5% of other ethnic group [9]. The electronic health records of the polyclinic showed 4,991 people with T2DM in 2013 (unpublished data).

### Participants

The eligible people were multi-ethnic Asian, aged 21 to 80 years, with known T2DM for a minimum of 2 years. Their diagnoses were verified with reference to their electronic health records (of affirmed oral glucose tolerance test or OGTT). They had at least one or more laboratory records of measurements of HbA1c, systolic and diastolic blood pressures and lipid profiles in the past 2 years or longer. These people would have undertaken annual diabetic eye screening within the polyclinic using digital photography of their retina to exclude diabetic retinopathy.

Excluded from the study were people with type 1 diabetes mellitus, newly diagnosed people with duration of T2DM of less than 2 years; absence of measurement of HbA1c, blood pressure or LDL-cholesterol or less in the past 2 years; absence of documented OGTT; with known congenital color blindness (achromatopsia) or (dyschromatopsia; intrinsic eye conditions and risk factors of ICV (diabetic retinopathy, cataract, glaucoma, cornea edema, optic neuritis or history of migraine, digoxin, aminoglycoside ingestion or recorded previous exposure to toxic chemicals) and existing cognitive disorders and psychiatric ailments, which may affect their ability to undertake the color vision screening test.

### Instrument

The Farnsworth D-15 instrument was selected for this study due to its relatively good precision and ease of administration [reliability index *K* = 0.96, and validity for qualitative classification *K* = 0.73–0.94,based on the Research & Technology Organization report of the North Atlantic Treaty Organization (NATO)] [10]. The original instrument developed by Farnsworth involves a color circle made from a blue ‘pilot’ or reference Munsell paper (B5/6) and 15 other colored (5/4) numbered disc of effective diameter about 12 mm. Each is mounted in a plastics dropper bottle cap, the color being visible through the aperture but relatively safe from soiling. The caps 1–15 are randomly arranged on the tray formed by the lid of their box and the pilot is placed at one end of the box base.

The participant chooses the color appearing to match the pilot color most closely and places it next to the pilot; he completes the sequence always matching the one he places last. He is not pressed for time and may change the order. The lid is closed, the box is inverted and on opening it the numbered bases of the caps are seen; they are recorded and a diagram is completed. The color arrangement made by the participant is drawn on a circular diagram representing the hues.

A normal trichromat correctly makes a sequence from 1 to 15. Protans, deutans and tritans make characteristic errors along the respective axes on the diagrams.

### Recruitment and color vision screening of the participants

The investigators screened potential participants for their enrolment eligibility on a case-encounter basis. Those who satisfied the inclusion and exclusion criteria were informed of the study protocol and their doubts were clarified before their endorsement of informed consent according to IRB approved procedure.

The research assistant interviewed the participants and filled up a questionnaire to capture information pertaining to their respective demographic and disease characteristics. The participants then undertook the color vision screening test using the Farnsworth D-15 instrument as described above. Color vision testing for all participants was done in the same room, beside the window with natural daylight illumination.

The screening test would separate them into two groups (1) strong/medium ICV (“ICV+”) or (2) mild ICV or normal color vision (“ICV-“). The type of defect emerged by comparing the crossover-lines to determine if they were parallel to the protan (red), deutan (green) or tritan (blue) color confusion axes. Detection of any of these anomalies would be regarded as “ICV+”. To ensure consistency of the primary outcome, the interpretation of the crossover lines was cross-checked between the two optometrists in the study team (KS and WFY). Ambiguous outcomes from the screening test were removed from the final analysis of the results.

Next, the research assistant retrieved the relevant clinical data and investigation results from the polyclinic electronic health records of the enrolled participants. Clinical data included the duration of T2DM (to the closest month), systolic and diastolic blood pressure, weight, body mass index, presence/absence of DM-related complications such as previous coronary, cerebral and peripheral vascular diseases, nephropathy (presence/absence of microalbuminuria or proteinuria). Treatment details comprised of the doses and types of oral hypoglycemic drugs, and/or types, doses and frequency of insulin therapy. Laboratory investigations included prior affirmed oral glucose tolerance test (OGTT), glycated hemoglobin (HbA1c) and fasting lipid profiles (Total cholesterol [TC], High-Density Lipoprotein-Cholesterol [HDL-C], Low-Density Lipoprotein-Cholesterol [LDL-C]) triglyceride [TG]. The mean and highest HbA1c, TC, HDL-C, LDL-C and triglycerides (in mg/dL) for current year (2013) and previous year (2012) would be used for the data analysis. These data were entered into an electronic spreadsheet. The data management officer from the institution department of research independently audited the data, rectified any error and anonymized the data set before handling it over to the biostatistician in the team for analysis.

### Pilot study

No precedent large scale ICV screening had been conducted in Singapore. A pilot study of 15 people with T2DM was carried out at the study site, which showed that 47% of them had ICV, as detected by the Farnsworth D-15 test (abbreviated as D-15 test) and 80% rated it favorably in its execution. The time taken to complete a color vision screening using this instrument ranged from 8 to 15 min.

### Sample size calculation

Based on the preliminary data from the pilot study, a conservative hypothesis was conceived that 47% of the respondents would present with ICV as defined by the Farnsworth D-15 test. In order to be able to prove this proportion with a 3% margin of error, we needed to recruit 1010 people.

### Statistical analysis

Participants were divided into two groups: those with ICV and those without ICV. Descriptive analysis was conducted to test the difference between the demographic groups using chi-square test and independent *t* test. Mann Whitney *U* test was performed for data on duration which were skewed. Fisher’s exact test was performed for the type of ICV, side of infected eye and visual acuity variables. The potential risk factors (variables which attained a *p* < 0.2 in the univariate analysis) were entered into the logistic regression analysis and analyzed for their association with ICV.

### Ethics approval

The study was approved by the SingHealth Centralized Institutional Review Board (CIRB reference 2012/926/E).

## Results

Amongst the 1008 recruited participants between Nov 2012 and Nov 2013, 128 were excluded due to incomplete clinical and laboratory data (such as absence of documented OGTT and no prior diabetic eye screening to exclude diabetic retinopathy). .A total of 31 (3.7%) participants were excluded as the results from the Farnsworth D15 test did not follow any specific pattern on second examination (performed on the same day), even after further instructions were given. Eventually 849 participants with complete data were analyzed.

Table 1 showed that in this study population, 22.3% of participants with T2DM but without diabetic retinopathy had ICV. Higher age was associated with ICV. Gender, ethnic groups and employment status were not significantly associated with ICV. There was no association with weight, height, body mass index, comorbidities such as hypertension, dyslipidemia, coronary, cerebrovascular and renal diseases. Amongst the prescribed diabetic medications, only Tolbutamide was associated with ICV. Compared to those without ICV, those with ICV had lower latest mean systolic blood pressure (126.7 vs 129.7 mmHg) and latest mean diastolic blood pressure (72.1 vs 73.9 mmHg) in 2013.

**Table 1:** Baseline characteristics of study population

	Total	ICV not present	ICV present	*p*-value
	849 (100.0)	660 (77.7)	189 (22.3)	-
Demographics				
Age, mean(SD)	57.4 (7.5)	57 (7.8)	59 (6.4)	<0.01*
Gender				0.54
Male	419 (49.4)	322 (76.8)	97 (23.2)	
Female	430 (50.6)	338 (78.6)	92 (21.4)	
Ethnic group				0.19
Chinese	459 (54.1)	345 (75.2)	114 (24.8)	
Malay	272 (32)	216 (79.4)	56 (20.6)	
Indian	104 (12.2)	87 (83.7)	17 (16.3)	
Others	14 (1.6)	12 (85.7)	2 (14.3)	
Highest Education Level attained				0.04*
Primary	190 (22.5)	139 (73.2)	51 (26.8)	
Secondary	468 (55.3)	361 (77.1)	107 (22.9)	
Diploma	93 (11)	79 (84.9)	14 (15.1)	
Tertiary	95 (11.2)	81 (85.3)	14 (14.7)	
Employment status				0.98
Unemployed	351 (41.3)	273 (77.8)	78 (22.2)	
Employed	498 (58.7)	387 (77.7)	111 (22.3)	
Weight (nearest kg)	73 (14.4)	73.3 (14.5)	72.1 (14.1)	0.32
Height (nearest cm)	161.7 (9.2)	161.8 (9)	161.5 (10.2)	0.72
BMI (nearest 0.1 decimal point)	27.9 (5)	28 (5)	27.7 (5)	0.57
Comorbidities				
Duration of Diabetes Mellitus (in years), median (IQR)	5 (3–9)	4 (3–8)	6 (3–10)	<0.01*
Hypertension				0.77
Yes	711 (83.7)	554 (77.9)	157 (22.1)	
No	138 (16.3)	106 (76.8)	32 (23.2)	
Duration of Hypertension (in years), median (IQR)	5 (2–10)	5 (2–10)	6 (2–10)	0.05
Dyslipidemia				0.56
Yes	794 (93.5)	619 (78)	175 (22)	
No	55 (6.5)	41 (74.5)	14 (25.5)	
Duration of Dyslipidemia (in years), median (IQR)	6 (3–9)	5 (3–9)	6 (3–9)	0.13
Coronary heart disease				0.49
Yes	121 (14.3)	97 (80.2)	24 (19.8)	
No	728 (85.7)	563 (77.3)	165 (22.7)	
Stroke/TIA				0.40
Yes	31 (3.7)	26 (83.9)	5 (16.1)	
No	818 (96.3)	634 (77.5)	184 (22.5)	
Renal disease (DM nephropathy)				0.40
Yes	52 (6.1)	38 (73.1)	14 (26.9)	
No	797 (93.9)	622 (78)	175 (22)	
Medications				
Subject is taking diabetic medication(s)				0.40
Yes	713 (84.3)	550 (77.1)	163 (22.9)	
No	133 (15.7)	107 (80.5)	26 (19.5)	
Duration of Diabetic medication (in years), median (IQR)	4 (1–8)	4 (1–7)	4 (1–8)	0.07
Sulphonylurea^a^				0.06
Yes	424 (50.1)	318 (75)	106 (25)	
No	422 (49.9)	339 (80.3)	83 (19.7)	
Glibenclamide				0.19
Yes	66 (7.8)	47 (71.2)	19 (28.8)	
No	780 (92.2)	610 (78.2)	170 (21.8)	
Glipizide				0.91
Yes	276 (32.6)	215 (77.9)	61 (22.1)	
No	570 (67.4)	442 (77.5)	128 (22.5)	
Gliclazide				0.73
Yes	35 (4.1)	28 (80)	7 (20)	
No	811 (95.9)	629 (77.6)	182 (22.4)	
Tolbutamide				<0.01*
Yes	42 (5)	24 (57.1)	18 (42.9)	
No	804 (95)	633 (78.7)	171 (21.3)	
Other sulphonylurea				0.11
Yes	23 (2.7)	21 (91.3)	2 (8.7)	
No	823 (97.3)	636 (77.3)	187 (22.7)	
Biguanide (Metformin)				0.42
Yes	667 (78.8)	522 (78.3)	145 (21.7)	
No	179 (21.2)	135 (75.4)	44 (24.6)	
Alpha-glucosidase inhibitor (Acarbose)				0.96
Yes	62 (7.3)	48 (77.4)	14 (22.6)	
No	784 (92.7)	609 (77.7)	175 (22.3)	
Insulin therapy^b^				0.57
Yes	104 (12.3)	83 (79.8)	21 (20.2)	
No	742 (87.7)	574 (77.4)	168 (22.6)	
Baseline insulin				0.12
Yes	83 (9.8)	70 (84.3)	13 (15.7)	
No	763 (90.2)	587 (76.9)	176 (23.1)	
Mixtard				0.05
Yes	23 (2.7)	14 (60.9)	9 (39.1)	
No	823 (97.3)	643 (78.1)	180 (21.9)	
Short-acting insulin				0.74
Yes	6 (0.7)	5 (83.3)	1 (16.7)	
No	840 (99.3)	652 (77.6)	188 (22.4)	
Clinical Profile				
Mean HbA1c -Yr 2012 (%)	7.2 (1.2)	7.3 (1.2)	7.1 (1)	0.25
Highest HbA1c -Yr 2012 (%)	6.8 (2.7)	6.8 (2.7)	6.7 (2.6)	0.54
Mean HbA1c -Yr 2013 (%)	7.4 (1.5)	7.4 (1.5)	7.4 (1.4)	0.91
Highest HbA1c -Yr 2013 (%)	7.0 (2.8)	6.9 (2.9)	7.4 (2.1)	0.01*
Mean LDL-Cholesterol- Yr 2012 (mg/dL)	92.8 (28.4)	92.5 (28.7)	93.7 (27.5)	0.63
Mean LDL-Cholesterol - Yr 2013 (mg/dL)	92.3 (28.6)	94.4 (29.1)	86.8 (26.5)	<0.01*
Mean Triglyceride - Yr 2012 (mg/dL)	133.4 (64.6)	134.9 (62.6)	128.4 (71.2)	0.93
Mean Triglyceride - Yr 2013 (mg/dL)	135.8 (72.5)	140.2 (74.4)	123.9 (66.2)	0.01*
Mean HDL-Cholesterol - Yr 2012 (mg/dL)	50.2 (13.9)	50.3 (14)	49.9 (13.3)	0.62
Mean HDL-Cholesterol - Yr 2013 (mg/dL)	50.9 (16.9)	49.9 (15.9)	53.6 (19.2)	0.02*
Mean Total Cholesterol - Yr 2012 (mg/dL)	169.6 (35.6)	169.3 (36.2)	170.4 (33.8)	0.25
Mean Total Cholesterol - Yr 2013 (mg/dL)	168.9 (37.9)	171.3 (39.6)	162.6 (32.4)	0.01*
Mean Systolic BP - Yr 2012 (mmHg)	128.4 (13)	128.5 (13.1)	128.4 (12.8)	0.72
Mean Systolic BP - Yr 2013 (mmHg)	129 (14)	129.7 (14.3)	126.7 (12.8)	0.02*
Mean Diastolic BP - Yr 2012 (mmHg)	73.6 (8.7)	73.7 (8.6)	73.3 (8.9)	0.72
Mean Diastolic BP - Yr 2013 (mmHg)	73.5 (8.9)	73.9 (9)	72.1 (8.5)	0.01*

*Abbreviations*: *ICV* impaired colour vision, *BMI* body mass index, *IQR* interquartile range, *HbA1c* glycated hemoglobin, *LDL* low-lipoprotein, *HDL* high-lipoprotein, *BP* blood pressure

^a^Sulphonylurea includes glibenclamide, glipizide, gliclazide, tolbutamine, and other sulphonylurea

^b^Insulin therapy includes any form including baseline, short-acting and mix insulin

*p < 0.05

Table 1: Participants with ICV were significantly associated with age and lower education; longer duration of diabetes (median duration: 6 years vs 4 years); highest HbA1c level in 2013 (mean 58 mmol/mol, or 7.4% vs 51 mmol/mol, or 6.9% for those without ICV); lower mean TC in 2013 (162.6 vs 171.3 mg/dL); higher HDL-C in 2013 (53.6 vs 49.9 mg/dL); lower mean LDL-C in 2013 (86.8 vs 94.4 mg/dL) and lower mean triglyceride in 2013 (123.9 vs 140.2 mg/dL).

Table 2 showed that more participants with ICV tended to have poorer vision beyond 6/12 in the affected eye. Tritanomaly was the commonest ICV in any eye, although higher number of affected participants had involvement of their right eyes.

**Table 2:** Visual acuity and Impaired Colour Vision (ICV)

Visual acuity	ICV not present	ICV present	
6/12 or better			<0.01*
Yes	658 (78.5)	180 (21.5)	
No	2 (18.2)	9 (81.8)	
6/18 or better			0.22
Yes	660 (77.8)	188 (22.2)	
No	0 (0)	1 (100)	
6/24 or better			-
Yes	660 (77.7)	189 (22.3)	
No	0 (0)	0 (0)	
	Right Only	Left Only	
Type of ICV			0.16
Protan	0 (0)	2 (100)	
Deutan	7 (70.0)	3 (30.0)	
Tritan	49 (64.5)	27 (35.5)	

*Abbreviations*: *ICV* impaired colour vision

*p < 0.05

Table 3: Logistic regression showed that ICV was associated with increased duration of T2DM with an odds ratio of 1.07 (95% CI: 1.02–1.12, *p* < 0.01); prescription of Tolbutamide (OR = 3.79, 95% CI: 1.65–8.74, *p* < 0.01) and lower mean systolic blood pressure in 2013 (OR = 0.98, 95% CI: 0.96–0.99, *p* = 0.01)

**Table 3:** Factors associated with presence of ICV using logistic regression

	OR (95% CI)	*p*-value
Age	1.04 (1.01–1.07)	0.01*
Highest Education Level		
Primary	1	-
Secondary	0.99 (0.6–1.61)	0.95
Diploma	0.53 (0.23–1.2)	0.13
Tertiary	0.71 (0.33–1.52)	0.38
Duration of DM	1.07 (1.02–1.12)	<0.01*
Visual acuity - Aided		
No	1	-
Yes	0.72 (0.48–1.06)	0.10
Tolbutamide		
No	1	-
Yes	3.79 (1.65–8.74)	<0.01*
Highest HbA1c (Yr 2013), mmol/L	1.04 (0.92–1.18)	0.51
Mean LDL-Cholesterol (Yr 2013), mmol/L	0.87 (0.65–1.17)	0.35
Mean Systolic BP (Yr 2013), mmHg	0.98 (0.96–0.99)	0.01*
Mean Diastolic BP (Yr 2013), mmHg	1.01 (0.98–1.03)	0.58
Mean Triglyceride (Yr 2013), mmol/L	0.78 (0.57–1.08)	0.14
Mean HDL-Cholesterol (Yr 2013), mmol/L	1.36 (0.87–2.13)	0.17

*Abbreviations*: *ICV* impaired colour vision, *HbA1c* glycated hemoglobin, *LDL* low-lipoprotein, *HDL* high-lipoprotein, *BP* blood pressure

*p < 0.05

## Discussion

This is by far the largest study to determine the prevalence of ICV amongst multi-ethnic Asian people with T2DM. One in five (22.3%) was detected to have ICV, predominantly tritanomaly, using the D-15 test. Earlier studies also showed the association between T2DM and the loss of discrimination along the blue-yellow axis in the presence of early diabetic retinopathy. People with diabetic retinopathy had been excluded in this study, yet there was increased incidence of tritanomaly [11, 12]. Nonetheless, there has been inconclusive evidence pertaining to the pathological pathways towards the development of tritanomaly. Tritan defects have been explained by higher susceptibility of short-wavelength cones (S-cones) in post-mortem examinations of the retina in a small study by Cho et al., and early yellowing of the lens in the diabetic eye [13]. Increasing evidence links it to primary neurodegenerative process of the retina that affects the colour vision of people with T2DM [14].

Previous studies by Andrade et al., Feitosa-Santana C et al., and Sun TS and Zhang MN showed that ICV could develop in the early stage of T2DM but did not specify the minimal period for the development of color vision deficiency [2–4]. The results of this study suggest that the risk of DM increases for each additional year after the onset of T2DM. People were more likely to develop ICV after 6 years. Thus age is associated with ACVI (OR = 1.04) with older patients often having longer duration of T2DM. As ICV impacts on daily activities and social life, the introduction of color vision screening for people with T2DM, possibly 6 years after its onset, could enhance the detection of ICV. Any screening program should be simple, affordable, easy to implement and provides opportunities for effective intervention which will improve the health outcomes of the affected people [15]. For the detection of ICV, screening using the D-15 test seemed feasible, as it was proven to be a simple and non-invasive procedure which could be completed in 15 min or sooner in this study.

Educating affected people should be an integral part of the screening program. As tritanomaly is the commonest ICV, patient education program should focus on strategies to empower them to cope with their activities involving blue-yellow color distinction. Research is needed to determine if such color vision screening is impactful on affected patients’ quality of life and its cost-effectiveness.

Previous studies showed a relationship between hyperglycemia, as reflected in glycated hemoglobin and ICV [2, 3]. Whilst there was no difference in the highest Hba1c between people with and without ICV in the first year (2012), the highest HbA1c increased amongst those with ICV in the 2nd year (2013). Nevertheless, ICV was not significantly associated with the mean HbA1c after the logistic regression.

Similarly, the systolic blood pressure and lipid profiles of people with ICV significantly improved in the 2nd year in terms of lower mean TC, LDL-C and triglycerides and higher mean HDL-C. This outcome contradicts the Okubo Study Report 1 and 2 in which ICV was associated with elevated LDL-C and systolic and diastolic blood pressures. However, the Okubo study included both healthy participants and those without T2DM and its cross-sectional method did not permit any observation of biochemical trend and progression of its participants clinical conditions [16, 17]. In the SNDREAMS- II 3rd Report, HDL-C was higher in the non-ICV group [5]. The authors concluded that HDL-C was a protective factor against ICV based on factor analysis but no chronological observation of trend was included in that study.

Thus, a remarkable finding in this study was the improvement in systolic blood pressure, glycemia and lipid status over a one-year period was associated with ICV. However this short retrospective study would be inappropriate to suggest any cause and effects. Such factors could reflect normal variations over the 1 year observation period. A randomized controlled trial would be a more appropriate study design to determine the effects of intensified control of blood pressure and glycemia in patients with diabetes mellitus on the development of ICV.

People treated with Tolbutamide were more than thrice as likely to be associated with ICV (OR = 3.79). It is one of the older sulphonylurea used in the treatment of T2DM in the polyclinics. According to the manual of ocular diagnosis and therapy, Tolbutamide, together with older sulphonylurea such as Chlorpropamide and Tolazamide, were listed to be associated with color vision defect [18]. In a case series report by Thomson DG et al., 4 people with 7 to 28 years of T2DM and treated with Tolbutamide, were found to develop ICV [19]. Two of them were detected to have Tritanomaly. Physicians can consider replacement of Tolbutamide with newer sulphonylurea medications, as a preventable measure to curb the development of ICV.

### Strength and limitations

Apart from the size of the study population, the study provided a rare insight into the change in clinical and biochemical parameters over a 2 year period. As a primary care study, the authors had put in a rigorous process to ensure that the participants were affirmed cases of T2DM based on international criteria for its diagnosis using the OGTT and to exclude those with diabetic retinopathy based on their annual digital retinal photography. The date of the OGTT was also used to compute the duration of the T2DM.

Thirty-one participants were excluded due to uncertain results of their screening tests. This could be due to subjects’ lapse of understanding of the instructions, thereby affecting the execution of the Farnsworth D-15 test. The inclusion of these 31 dubious results might potentially skew the results. These ambiguous results were deliberately excluded from the data analysis. The exclusion of these participants was unlikely to result in a selection bias, as only 3.7% of the total recruited participants were excluded.

The study has its limitations. Farnsworth D15 test was used to screen and identify those with ICV. Patients with mild ICV or diffuse losses in color vision might potentially be misclassified as individuals with normal color vision due to the lack of discrimination power of the Farnsworth D15 instrument.

Furthermore, the duration of co-morbidities such as those relating to vascular diseases were excluded in the data analysis as such data based on participants’ self-reporting in the electronic health records may not be reliable.

## Conclusion

22.3% of the participants with T2DM had ICV. Tritanomaly was the predominant type of ICV and affected both eyes. Participants with ICV were associated with lower education status, longer duration of T2DM, prescribed with Tolbutamide and had lower systolic blood pressure is subsequent year. The results suggest that color vision screening of people for ICV after they have T2DM for 6 years or longer, but such a program awaits further feasibility and cost-effectiveness evaluation.

## References

[1] Shoji T, Sakurai Y, Sato H, Chihara E, Takeuchi M (2011). Do type 2 diabetes patients without diabetic retinopathy or subjects with impaired fasting glucose have impaired colour vision? The Okubo color study report. Diabet Med.

[2] Andrade LCO, Souza GS, Lacerda EMC, Nazima MT, Rodrigues AR, Otero LM (2014). Influence of retinopathy on the achromatic and chromatic vision of patients with type 2 diabetes. BMC Ophthalmol.

[3] Feitosa-Santana C, Paramei GV, Nishi M, Gualtieri M, Costa MF, Ventura DF (2010). Color vision impairment in type 2 diabetes assessed by the D-15d test and the Cambridge Colour Test. Ophthalmic Physiol Opt.

[4] Sun T, Zhang M (2012). Characters of contrast sensitivity in diabetic patients without male retinopathy. Zhonghua Yan Ke Za Zhi [Chin J Ophthalmol].

[5] Gella L, Raman R, Kulothungan V, Pal SS, Ganesan S, Sharma T. Impairment of Colour Vision in Diabetes with No Retinopathy: Sankara Nethralaya Diabetic Retinopathy Epidemiology and Molecular Genetics Study (SNDREAMS- II, Report 3). PLoS ONE [Internet]. 2015 Jun 8 [cited 2016 May 16];10(6). Available from: http://journals.plos.org/plosone/article?id=10.1371/journal.pone.0129391. 10.1371/journal.pone.0129391 PMC4460124 26053017

[6] Trento M, Passera P, Trevisan M, Schellino F, Sitia E, Albani S (2013). Quality of life, impaired vision and social role in people with diabetes: a multicenter observational study. Acta Diabetol.

[7] Phan TP, Alkema L, Tai ES, Tan KHX, Yang Q, Lim W-Y, et al. Forecasting the burden of type 2 diabetes in Singapore using a demographic epidemiological model of Singapore. BMJ Open Diabetes Res Care [Internet]. 2014 Jun 11 [cited 2016 May 16];2(1). Available from: https://www.ncbi.nlm.nih.gov/pubmed/25452860 10.1136/bmjdrc-2013-000012 PMC4212579 25452860

[8] Sng Q. Primary Care Survey 2010. Profile of Primary Care Patients. [Internet]. [cited 2015 Oct 10]. Available from: https://www.moh.gov.sg/content/dam/moh_web/Publications/Information%20Papers/2011/Primary%20Care%20Survey%202010%20-%20Profile%20of%20Primary%20Care%20Patients.pdf

[9] Department of Statistics, Ministry of Trade & Industry, Republic of Singapore. Census of population 2010 [Internet]. Resident Population by Planning Area, Age Group and Sex. [cited 2016 May 16]. Available from: https://www.singstat.gov.sg/docs/default-source/default-document-library/publications/publications_and_papers/cop2010/census_2010_advance_census_release/c2010acr.pdf

[10] RESEARCH AND TECHNOLOGY ORGANIZATION, BP 25, 7 RUE ANCELLE, F-92201 NEUILLY-SUR-SEINE CEDEX, FRANCE. Operational Colour Vision in the Modern Aviation Environment [Internet]. France; [cited 2016 May 16] p. 176. Report No.: 16. Available from: http://www.dtic.mil/dtic/tr/fulltext/u2/a390357.pdf

[11] Fong DS, Barton FB, Bresnick GH (1999). Impaired color vision associated with diabetic retinopathy: early treatment diabetic retinopathy study report No. 15. Am J Ophthalmol.

[12] Ayed S, Jeddi A, Kallal Z (1990). Diabetes and color vision disorder detected by the Farnsworth 100 Hue test. Diabetic dyschromatopsia. J Fr Ophtalmol.

[13] Cho NC, Poulsen GL, Ver Hoeve JN, Nork TM (2000). Selective loss of S-cones in diabetic retinopathy. Arch Ophthalmol.

[14] Zhang X, Wang N, Barile GR, Bao S, Gillies M (2013). Diabetic retinopathy: neuron protection as a therapeutic target. Int J Biochem Cell Biol.

[15] Wilson JMG, Jungner G (1968). Principles and practice of screening for disease. J R Coll Gen Pract.

[16] Shoji T, Sakurai Y, Chihara E, Nishikawa S, Omae K (2009). Reference intervals and discrimination values of the lanthony desaturated D-15 panel test in young to middle-aged Japanese army officials: the Okubo color study report 1. Eye Lond Engl.

[17] Shoji T, Sakurai Y, Sato H, Chihara E, Ishida M, Omae K (2010). Serum low-density lipoprotein cholesterol level is strong risk factor for acquired color vision impairment in young to middle-aged Japanese men: the Okubo color study report 2. Atherosclerosis.

[18] DeborahPavan-Langston. Manual of Ocular Diagnosis and Therapy, 6th edition. Clin Exp Optom. 2009;92(5):464.

[19] Thompson DG, Howarth F, Taylor H, Levy IS, Birch J (1979). Defective colour vision in diabetes: a hazard to management. Br Med J.

